# *AceE* affects the optimum growth and biofilm formation of *Mycobacterium tuberculosis* via cell wall lipid remodeling

**DOI:** 10.1128/msystems.01732-25

**Published:** 2026-04-20

**Authors:** Suting Chen, Jifang Zheng, Qiang Liu, Tianlu Teng, Ziyi Yang, Fengmin Huo, Yi Xue, Liang Li, Hairong Huang

**Affiliations:** 1 National Clinical Laboratory on Tuberculosis, Beijing Key Laboratory for Drug-resistant Tuberculosis Research, Beijing Chest Hospital, Capital Medical University, Beijing Tuberculosis and Thoracic Tumor Institute 12517, Beijing, China; 2 Department of Respiratory and Critical Care Medicine, Beijing Chest Hospital, Capital Medical University, Beijing Tuberculosis and Thoracic Tumor Institute 12517, Beijing, China; UiT Norges Arktiske Universitet Arctic Centre for Sustainable Energy, Tromsø, Norway

**Keywords:** *aceE*, *Mycobacterium tuberculosis*, lipid metabolism, growth, pathogenicity

## Abstract

Central carbon metabolism is fundamental to the growth and virulence of *Mycobacterium tuberculosis* (Mtb). The *aceE* gene encodes the E1 subunit of the pyruvate dehydrogenase complex that irreversibly converts pyruvate to acetyl-CoA. There, we investigated the impact of *aceE* inactivation on the physiological metabolism of Mtb. An *aceE* deletion in Mtb H37Rv was constructed, and the mutant was compared to wild type (WT) by *in vitro* growth assays, colony morphology, biofilm formation, stress tolerance, lipidomics, RNA-seq, and BALB/c mouse infection. Mtb Δ*aceE* exhibited markedly slower planktonic growth, smooth colony morphology, impaired biofilm formation, and was more sensitive to acid, NaNO_2_, and some commonly used antibiotics. Lipidomics revealed global depletion of mycolic acids and major phospholipids in Mtb Δ*aceE* mutant. Transcriptome analysis showed downregulation of TCA cycle and fatty-acid biosynthetic genes. Two weeks after intravenous infection, lung bacillary loads were 0.6 log10 lower for Δ*aceE* than in WT, and spleen enlargement was absent, yet the mutant persisted, and pathology was otherwise comparable to WT. AceE is required for robust lipid anabolism, biofilm formation, and maximal growth of Mtb *in vitro*, especially on carbohydrate-based media, but its absence only modestly attenuates acute virulence in mice, underscoring the pathogen’s metabolic plasticity during infection.

**IMPORTANCE:**

The present study demonstrated that the *aceE* gene, a crucial enzyme that links glycolysis and the TCA cycle, plays a vital role in regulating the normal physiological metabolism of *Mycobacterium tuberculosis* (Mtb). The *aceE* gene not only aids in the bacteria’s energy metabolism but also promotes lipid synthesis, forming a thicker cell wall that helps Mtb resist various intracellular stresses, further favoring its survival within the host cells. During *in vivo* survival, the increased expression of the *aceE* gene in the virulent Mtb H37Rv strain may enhance the conversion of pyruvate into acetyl-CoA, thereby providing more precursor materials for the synthesis of lipids and amino acids.

## INTRODUCTION

Tuberculosis (TB), caused by *Mycobacterium tuberculosis* (Mtb), continues to be a significant cause of death from an infectious agent. According to the World Health Organization (WHO), one-fourth of the world’s population is infected with Mtb, and in 2023, an estimated 1.25 million HIV-negative TB deaths occurred globally (1). Mtb’s ability to evade immune surveillance and achieve long-term survival in the body makes it a formidable foe. In about 90% of infected hosts with competent immune responses, Mtb decreases its metabolic rate, transforms into a persister, and ultimately causes latent infection (2, 3). This pathogen is a master at sensing changes in the external environment and entering a long-term dormant state, rendering it one of the most successful intracellular pathogens (4–6). The co-expression of multiple virulence genes, including those involved in intermediate metabolism, cell wall synthesis, oxidative/nitrosative stress, and signal transduction, has been linked to maintaining Mtb’s persistent infection (7–10). Despite this, the whole gene regulatory network underlying Mtb persistence remains unclear.

Central carbon metabolism (CCM) is the enzymatic transformation of carbon in organisms through processes such as glycolysis, the pentose phosphate pathway, the citric acid cycle, the glyoxylate shunt, the methylcitrate cycle, and gluconeogenesis (11, 12). CCM provides energy to organisms through redox reactions and also produces essential precursor substances for biosynthesis. Some enzymes involved in CCM have been identified as important virulence factors of Mtb (13–20). These enzymes play important roles in the metabolic processes and protect pathogens from immune surveillance by participating in antioxidant defense (15–20). Pyruvate dehydrogenase (PDH) in Mtb is an enzyme complex (consisting of E1, E2, and E3 enzymes) that catalyzes the conversion of pyruvate to acetyl-CoA. This process links glycolysis (where pyruvate is produced) and the TCA cycle (where acetyl-CoA is used as a starting reactant) through pyruvate decarboxylation and oxidation. Acetyl-CoA is an important intermediate metabolite that participates in the TCA and glyoxylate cycles, and it provides a carbon source for synthesizing mycolic acid and lipids required for the Mtb cell wall (21).

Studies have shown that the transcription of the three PDH genes is independent of Mtb, allowing them to perform more complex functions independently (22). The E2 and E3 components, encoded by *dlaT* and *lpd*, respectively, are associated with the persistence of Mtb infection. A deficiency in the *dlaT* gene results in significantly slower growth of the mutant compared to the WT Mtb in the standard medium, and it is highly susceptible to nitrogen reaction intermediates (23). The *dlaT*-deficient mutant is also attenuated in infected mice (24). Selective inhibitors of the *dlaT* gene can kill nonreplicated Mtb, suggesting an association with Mtb persistence *in vivo*. Additionally, the *lpd*-deficient Mtb strain was attenuated in both competent and immunodeficient mice. The *lpd* coding protein, which also serves as the E1 component of peroxynitrite reductase/peroxidase, was identified to help Mtb resist host reactive nitrogen intermediates (15). Our previous study found that inactivation of the *aceE* gene leads to defects in the mycolic acid synthesis of alpha-MA and epoxy-MA in *M. smegmatis*, resulting in morphological changes in bacteria and defects in biofilm formation (25). However, the role of the *aceE* gene, which encodes the E1 component of the pyruvate dehydrogenase complex in Mtb, has not been thoroughly studied. To address this, we created a Δ*aceE* mutant in Mtb H37Rv using homologous recombination to study how deficiency of this key rate-limiting enzyme in pyruvate metabolism affects the physiological metabolism and pathogenicity of Mtb.

## MATERIALS AND METHODS

### Construction of the Δ*aceE* mutant in Mtb H37Rv

The left- and right-flanking sequences of the *aceE* gene used for homologous recombination were amplified from the Mtb genome using gene-specific primers listed in Table S1 (VLL and VLR; VRL and VRR). The *Van91*I-digested PCR product was cloned into p0004s to generate p0004s-Δ*aceE*. The *Pac*I-digested p0004s-Δ*aceE* plasmids were then cloned into the phAE159 phasmid to generate the allelic exchange phasmid phAE159-Δ*aceE*. The temperature-sensitive phasmid was electroporated into *M. smegmatis* mc^2^155 and cultured at 30°C to obtain a high titer of phages. The phages were transduced into the Mtb H37Rv, and knockout candidates were selected on 7H10 agar plates supplemented with hygromycin (Hyg). Deletion of *the aceE* gene was confirmed by PCR amplification using multiple pairs of primers (Table S1). The complement strain overexpressing the *aceE* gene in the Δ*aceE* strain (Δ*aceE::aceE*) was constructed using the pMV361 plasmid. *Escherichia coli* DH5a and HB101 strains used for gene cloning were grown at 37°C using LB broth and LB agar supplemented with appropriate antibiotics. *M. smegmatis* mc^2^155 and Mtb H37Rv were grown in 7H9 broth medium or on 7H10 agar supplemented with 10% OADC, 0.05% (vol/vol) Tween-80, and 0.5% (vol/vol) glycerol.

### Bacterial growth

Mtb strains were grown into a mid-log phase and adjusted to an OD_600_ of 0.6. The bacterial solution was subsequently diluted 100 times with saline, then inoculated in 7H9 broth medium with 10% OADC and 0.05% (vol/vol) Tween-80 and 0.5% (vol/vol) glycerol, and cultured at 37°C. The OD_600_ value and bacterial numbers were measured at different time points. The colony morphology was observed and photographed after three weeks of incubation at 37°C. For culturing Mtb on single carbon sources, a base of 7H9 liquid medium with 0.05% tyloxapol was supplemented with either glycerol (0.4%), glucose (0.4%), sodium butyrate (0.2%), or oleic acid (0.06%). The bacteria in the logarithmic growth stage were washed once with PBS and resuspended in each medium. The initial bacterial concentration was adjusted to an OD_600_ of 0.06. The bacteria were incubated at 37°C, and OD_600_ was measured weekly.

### Biofilm formation assay

Bacteria in the logarithmic growth phase were first washed once with phosphate-buffered saline (PBS) and then re-suspended in a 7H9 basic medium (without Tween-80), to which OADC (oleic acid, albumin, dextrose, and catalase) or various unique carbon sources were added. The initial concentration of bacteria should be adjusted to an OD_600_ at a final value of 0.06. The cultures should be incubated for 4 weeks. During this time, whether the bacteria grew normally and the presence of biofilm formation at the gas-liquid interface or along the tube walls were checked.

For biofilm quantification (26, 27), the culture medium was carefully removed, whereas the remaining biofilm was rinsed with normal saline. 0.1% crystal violet was added for biofilm staining, and 95% ethanol was added to separate the crystal violet-attached biofilm. 100 microliters of the separation liquid was absorbed and transferred to a new 96-well plate, and the OD_570_ value was determined.

### Analysis of *in vitro* survival under different stress conditions

Mtb strains were grown to a mid-log phase in the 7H9 medium. The acidified 7H9 medium was adjusted using HCl. H37Rv and Δ*aceE* mutants were sub-cultured in neutral 7H9 medium (pH ~6.7) or acidified 7H9 medium (pH ~5.5) at 37°C, and the OD_600_ value and bacterial numbers were measured at different time points. H37Rv and Δ*aceE* mutants were treated with 1% SDS for 1 and 4 h. For the hydrogen peroxide stress test, strains were treated with 0~20 mM H_2_O_2_ for 4 h. In addition, for the reaction nitrogen stress test, strains were treated with 0~5 mM NaNO_2_ for 4 d. After treatment, the serial dilutions were cultured on 7H10 agar containing 10% OADC, whereas the number of colony-forming units (CFU) was enumerated 3 weeks later.

### Anti-TB drug susceptibility assays

Several antibiotics were used in this study, including isoniazid (INH), rifampicin (RFP), ethambutol (EMB), streptomycin (STR), amikacin (AMK), capreomycin (CMP), and levofloxacin (LFX). H37Rv and Δ*aceE* mutants were harvested in mid-log phase and adjusted to an OD_600_ of 0.06. Then the bacteria were diluted 100-fold and exposed to the following drugs with each concentration of INH 0.025 µg/mL, RFP 0.1 μg/mL, EMB 1.25 µg/mL, STR 0.5 µg/mL, AMK 0.125 µg/mL, CMP 0.5 µg/mL, and LFX 0.5 µg/mL. The culture was transferred to the MGIT960 culture tubes. The culture-positive detection time of each culture tube was monitored and reported by the MGIT960 system.

### Drug tolerance assay

Bacteria were adjusted to a concentration of 1 × 10^8^ CFU/mL and were exposed to RFP (0.5, 2, and 10 μg/mL) or LFX (2, 10, and 50 μg/mL) for 7, 14, and 21 days, respectively. After treatment with these antibiotics, CFU was determined using a 7H10 agar plate containing 10% OADC.

### Macrophage infection

The human monocyte cell line THP-1 was cultured in RPMI1640 medium (Invitrogen) supplemented with 2 mM glutamine, 10% (vol/vol) heat-inactivated fetal bovine serum (FBS; Gibco), 100 U/mL penicillin, and 100 µg/mL streptomycin (Invitrogen). THP-1 cells were seeded at 1 × 10^6^ cells per well in 12-well tissue culture plates and stimulated with 100 µg/mL phorbol 12-myristate 13-acetate (PMA, Sigma-Aldrich) for 48 h. Macrophages were infected with H37Rv or Δ*aceE* mutant at a multiplicity of infection (MOI) value of 1:1 or 1:10 at 37°C with CO_2_ for 3 h. Extracellular bacilli were removed by washing cells three times with PBS, and the cells were cultured in fresh complete medium. After 24 h of infection, the infected macrophages were washed with PBS and lysed with 0.05% SDS. The cell lysates were serially diluted 10-fold with 0.05% Tween-80 and then spotted on 7H10 agar supplemented with 10% OADC.

### Mice infection

The mycobacteria in the log phase were collected and washed once with 7H9 medium that did not contain antibiotics. The cell suspension was adjusted to an OD_600_ of 0.6, corresponding to 1 × 10^8^ CFU/mL. The bacterial suspensions were drawn through a 27 G needle and diluted with the saline solution to achieve a final concentration of 1 × 10^7^ CFU/mL before the challenge. Female BALB/c mice, aged 6–8 weeks, were housed in a biosafety level-3 laboratory and were challenged intravenously with 0.1 mL of the bacterial suspension. Three or four mice per group were sacrificed, and their lungs and spleen were aseptically isolated at 2-, 6-, and 12 weeks post-infection. The lungs and spleen were divided into two sections and weighed separately. Half of each was fixed with formalin for hematoxylin-eosin (HE) staining, while the other half was used for CFU enumeration after homogenization.

### Electron microscopy

For the scanning electron microscopy (SEM), mid-log growth phase bacteria were harvested, washed twice with PBS, and fixed using ice-cold 2.5% glutaraldehyde. After rinsing thrice with PBS, the samples were postfixed with 1% OsO_4_ (Ted Pella Inc.) for 2 h. The samples were then dehydrated through a graded series of ethanol up to 100% and transferred to acetone. The cells underwent critical point drying, were applied to adhesive carbon film, and were subsequently examined using a HITACHI SU8100 scanning electron microscope. For the transmission electron microscopy (TEM), the dehydrated cells were embedded with 812 embedding resin (SPI). Ultra-thin sections were prepared on an ultra-thin microtome (Leica) and were stained first with 2% uranyl acetate in saturated alcohol, followed by 2.6% lead citrate. Grids were viewed on a HITACHI HT7800/HT7700 transmission electron microscope, from which digital images were captured. Cell wall thickness was measured using Image-Pro Plus 6.0 (Media Cybernetics, Inc., Rockville, MD, USA). Bacterial cells from each group were randomly selected for this measurement, ensuring that the number of measures in each group was no less than 15.

### Analysis of the lipidome of Mtb

Logarithmic growth bacteria were collected and washed with pre-cooled PBS twice, and the supernatant was discarded by centrifugation. Ice-precooled methanol (0.5 mL) was added and mixed with the bacteria. The suspension was transferred to a new tube, and 0.25 mL ice-precooled chloroform was added. The sample suspension was stored at –80°C until analysis. Lipidome analysis was performed via liquid chromatography/mass spectrometry (LC/MS) by Lipidall Technologies Company Limited (Jiangsu, China).

### RNA sequencing

RNA sequencing was performed by Shanghai Biotechnology Corporation (Shanghai, China). Bacteria were cultured in the log phase and washed twice with PBS. Total RNA was extracted using TRIzol reagent (Invitrogen) according to the manufacturer’s instructions. Qualified total RNA was further purified by RNAClean XP Kit (Beckman Coulter, Inc., Kraemer Boulevard, Brea, CA, USA) and RNase-Free DNase Set (QIAGEN, GmbH, Germany). The cDNA library was generated using a VAHTS Universal V6 RNA-seq Library Prep Kit (Vazyme) for Illumina and sequenced on an Illumina system (Illumina). High-quality reads were mapped to the Mtb H37Rv reference genome, and the expression level of each gene was calculated as kilobases of exon model per million mapped reads. Several differentially expression genes with high fragments per kilobase of exon model per million mapped fragments (FPKM) values were randomly selected for validation by qRT-PCR using a qRT-PCR kit (TIANGEN, Beijing, China), and the *rrs* gene was used as an internal control. Relative expression levels were calculated as 2^–ΔΔCt^.

### Biosafety statement

All work with live Mtb was conducted under BSL-3 conditions in accordance with the national guideline WS 589-2018 (China). Infections were performed inside a Class II biosafety cabinet; waste was decontaminated by autoclaving (121°C, 30 min) or chemical disinfection with 5% phenol before disposal.

## RESULTS

### The *aceE* gene knockout affects the growth phenotype of Mtb *in vitro*

**Fig 1:**
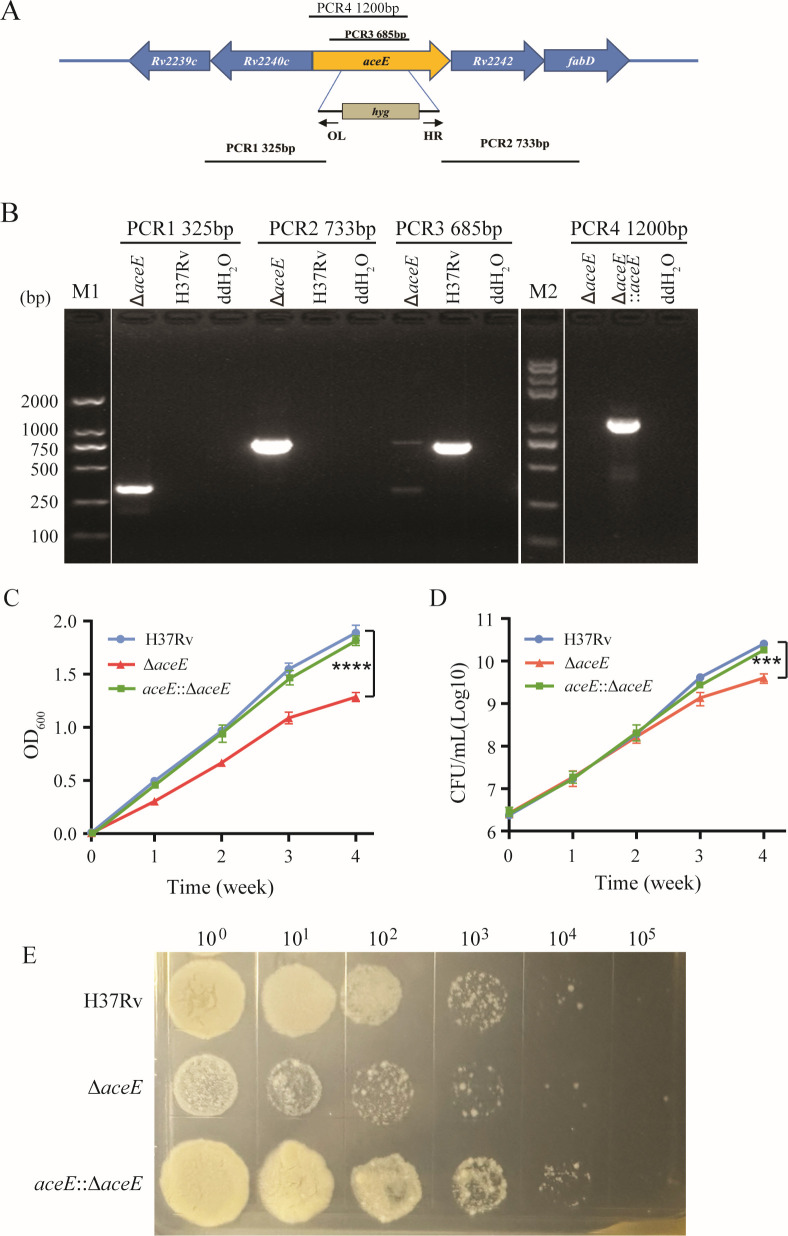
Comparison of bacterial growth between Mtb H37Rv wild-type (WT) strain and Mtb Δ*aceE* mutant strain. (**A**) Construction of *aceE* gene knockout mutant by homologous recombination elements containing hygromycin resistance gene (Hyg). (**B**) The deletion of the *aceE* gene and the complemented strain were identified by PCR using multiple pairs of primers. (**C**) Bacterial growth morphology on 7H10 and growth curve in 7H9 broth. (**D**) Growth curves in liquid 7H9 medium were plotted. (**E**) Growth morphology of Mtb H37Rv WT and Mtb Δ*aceE* mutant on solid 7H10 medium. The difference between each group’s growth in the 4th week was statistically analyzed using one-way ANOVA corrected by Tukey. ****, *P* < 0.0001; ***, *P* < 0.001. Mtb H37Rv WT strain: H37Rv; Mtb Δ*aceE* mutant: Δ*aceE*; Overexpressing *aceE* gene in Mtb Δ*aceE* mutant: Δ*aceE::aceE*.

The constructed homologous recombinant plasmid was transformed into the Mtb H37Rv WT strain, and the deletion of the *aceE* gene (Mtb Δ*aceE*) was confirmed through multiple PCR fragments (Fig. 1A and B). Similar to the *M. smegmatis aceE*-Tn mutant, the Mtb Δ*aceE* mutant exhibited slower replication compared to the Mtb H37Rv WT strain after 2 weeks of growth in 7H9 complete medium, as evidenced by bacterial turbidity and CFU counts (Fig. 1C and D). Additionally, the Mtb Δ*aceE* mutant grows significantly slower than the MtbH37Rv WT strain when grown on a solid 7H10 medium (Fig. 1E). In contrast, the complement strain grows similarly to the MtbH37Rv WT strain.

### The Mtb Δ*aceE* mutant exhibits abnormal cell morphology and biofilm formation defects

**Fig 2:**
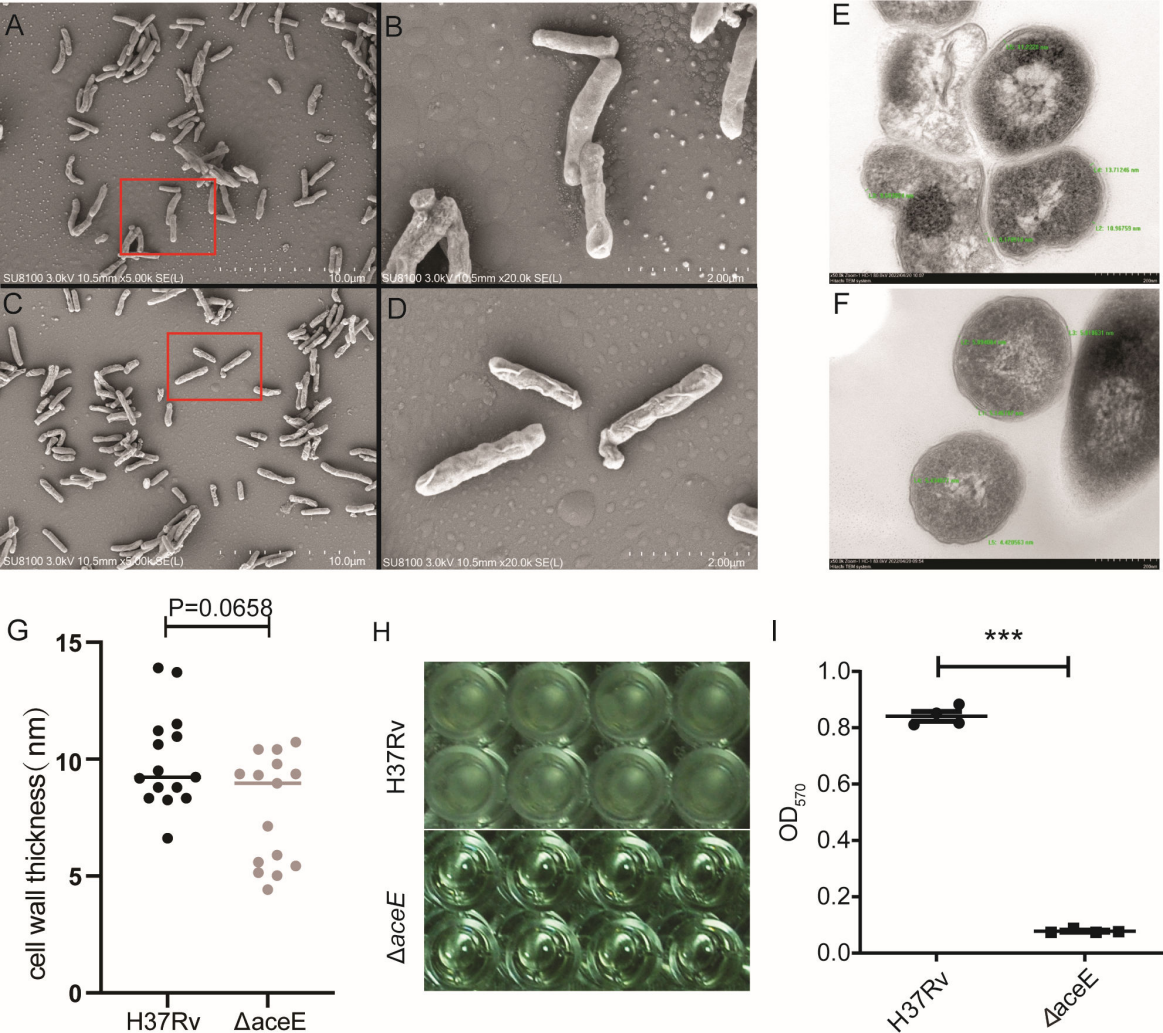
Bacterial morphology under SEM and TEM and its biofilm formation. SEM images of WT (**A and B**) and Mtb Δ*aceE* mutant (**C and D**) strains were collected with the magnification of 5,000× and 20,000×. TEM images of H37Rv WT (**E**) and Mtb Δ*aceE* mutant (**F**) strains were collected with a magnification of 50,000×. (**G**) Cell wall thickness of WT and Mtb Δ*aceE* mutant strains was measured using the Image-Pro Plus 6.0 software. The quantitative results regarding the cell wall were obtained from multiple measurements taken from three randomly selected fields of view for both the WT strain and the MtbΔaceE strain. The difference between each group was statistically analyzed using a Mann-Whitney *U* test. (**H**) Biofilm structure was formed at the gas-liquid interface of the culture tube inoculated with Mtb H37Rv wild-type strain, while the ΔaceE mutant strain could not form biofilm structure. (**I**) Quantitative results of crystal violet staining. Data show mean ± SD of one representative experiment performed in sextuplicate. The difference between each group was statistically analyzed using an unpaired *t*-test. *, *P* < 0.05; ***, *P* < 0.001. Mtb H37Rv WT strain: H37Rv; Mtb Δ*aceE* mutant: Δ*aceE*.

Since the Mtb Δ*aceE* mutant exhibits dramatically attenuated growth, we examined whether the morphology of individual mutant cells differs from that of WT cells. Under the scanning electron microscope, the WT cells display a relatively smooth cell wall surface, giving them a healthy and vigorous appearance (Fig. 2A and B). In contrast, the Mtb Δ*aceE* cells exhibit more wrinkles on their surface (Fig. 2C and D), suggesting some defects in their cell wall. Transmission electron microscopy, used for examining the cell morphology and the cell wall architecture, further showed that the Mtb Δ*aceE* cells possessed a slightly thinner cell wall than the WT cells (*P* = 0.0658) (Fig. 2E through G). To investigate the impact of *aceE* gene inactivation on the biofilm formation of Mtb, we assessed biofilm formation in the WT strain and the *aceE* knockout strain in complete 7H9 medium without Tween-80. The results of biofilm formation analysis in Mtb also showed that the Mtb Δ*aceE* mutant could not form a biofilm in the gas-liquid interface (Fig. 2H and I). In contrast, the MtbH37Rv WT strain formed robust biofilms.

### The Mtb Δ*aceE* mutant was more sensitive to acidic medium and NaNO_2_

**Fig 3:**
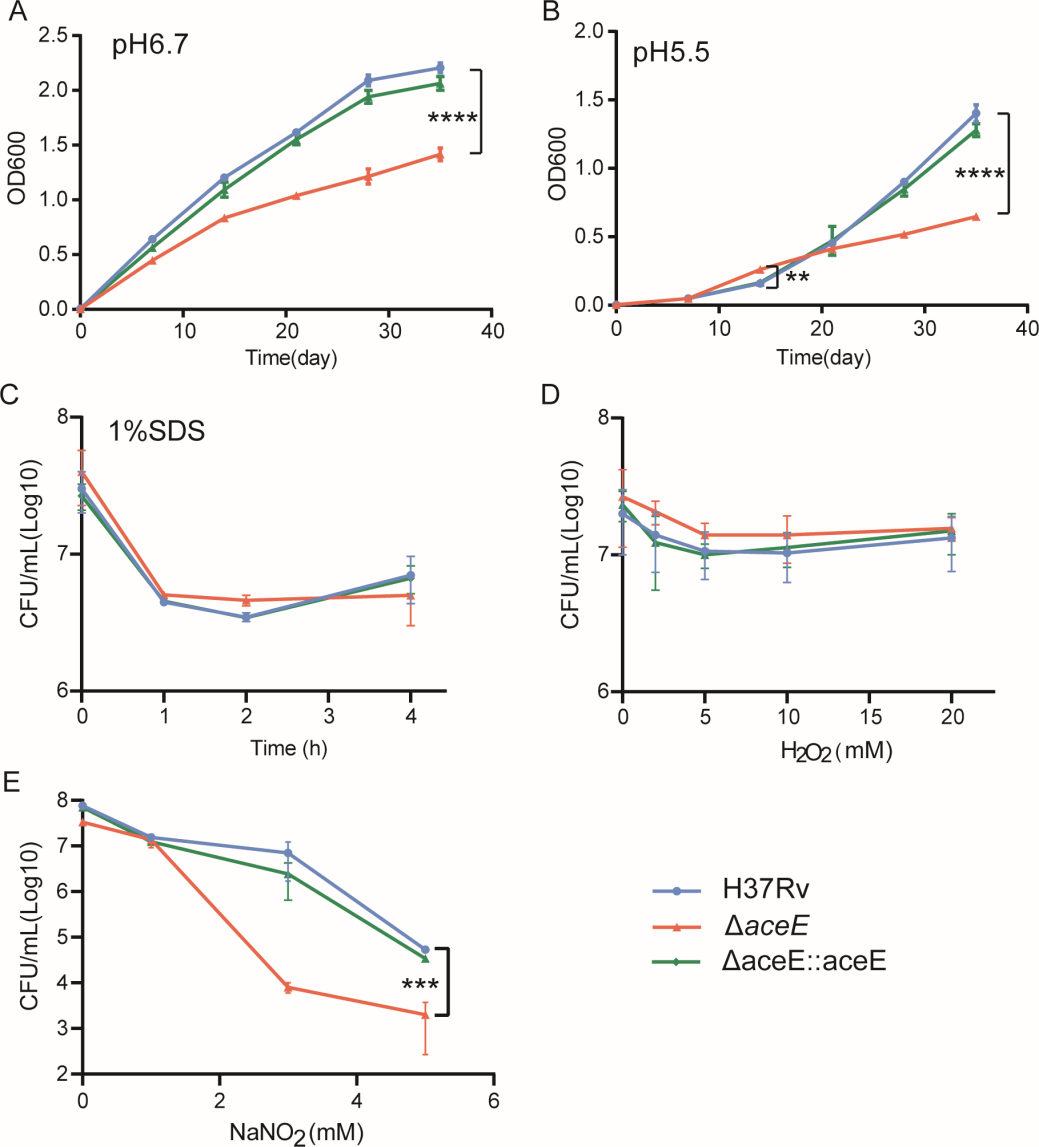
Effect of *aceE* gene inactivation on the growth of Mtb in acid 7H9 medium and under various chemical stress. Bacterial growth in acid 7H9 medium. The growth curves of Mtb H37Rv WT and Mtb Δ*aceE* mutant in neutral (pH 6.7) 7H10 medium (**A**) and in acidic (pH 5.5) 7H9 medium (**B**) were measured. (**C**) Bacteria were treated with 1% SDS for different time periods, and the survival of bacteria was counted by CFU. (**D**) Bacteria were treated with serial concentrations of H_2_O_2_, and the CFU were measured after 24 h; (**E**) Bacteria were treated with different concentrations of NaNO_2_ for 3 days. Data are means ± SD of triplicate cultures and are representative of two independent experiments. The difference between each group at each condition was statistically analyzed using an unpaired *t*-test. ***, *P* < 0.001; ****, *P* < 0.0001. Mtb H37Rv WT strain: H37Rv; Mtb Δ*aceE* mutant: Δ*aceE*; overexpressing *aceE* gene in Mtb Δ*aceE* mutant: Δ*aceE::aceE*.

Since the Mtb Δ*aceE* mutant is defective in cell wall and biofilm formation, we hypothesized that the *aceE*-deficient mutant would be sensitive to some stress conditions. The growth of WT, the mutant strain, and the complement strain in an acidic medium, SDS, H_2_O_2_, and NaNO_2_ was compared (Fig. 3). The Mtb Δ*aceE* mutant was more sensitive to acidic medium (Fig. 3B) and NaNO_2_ treatment (Fig. 3E). However, there was no significant difference between the WT and mutant when treated with SDS and H_2_O_2_ (Fig. 3C and D).

### Inactivation of the *aceE* gene affects the tolerance of Mtb to antibiotics

**Fig 4:**
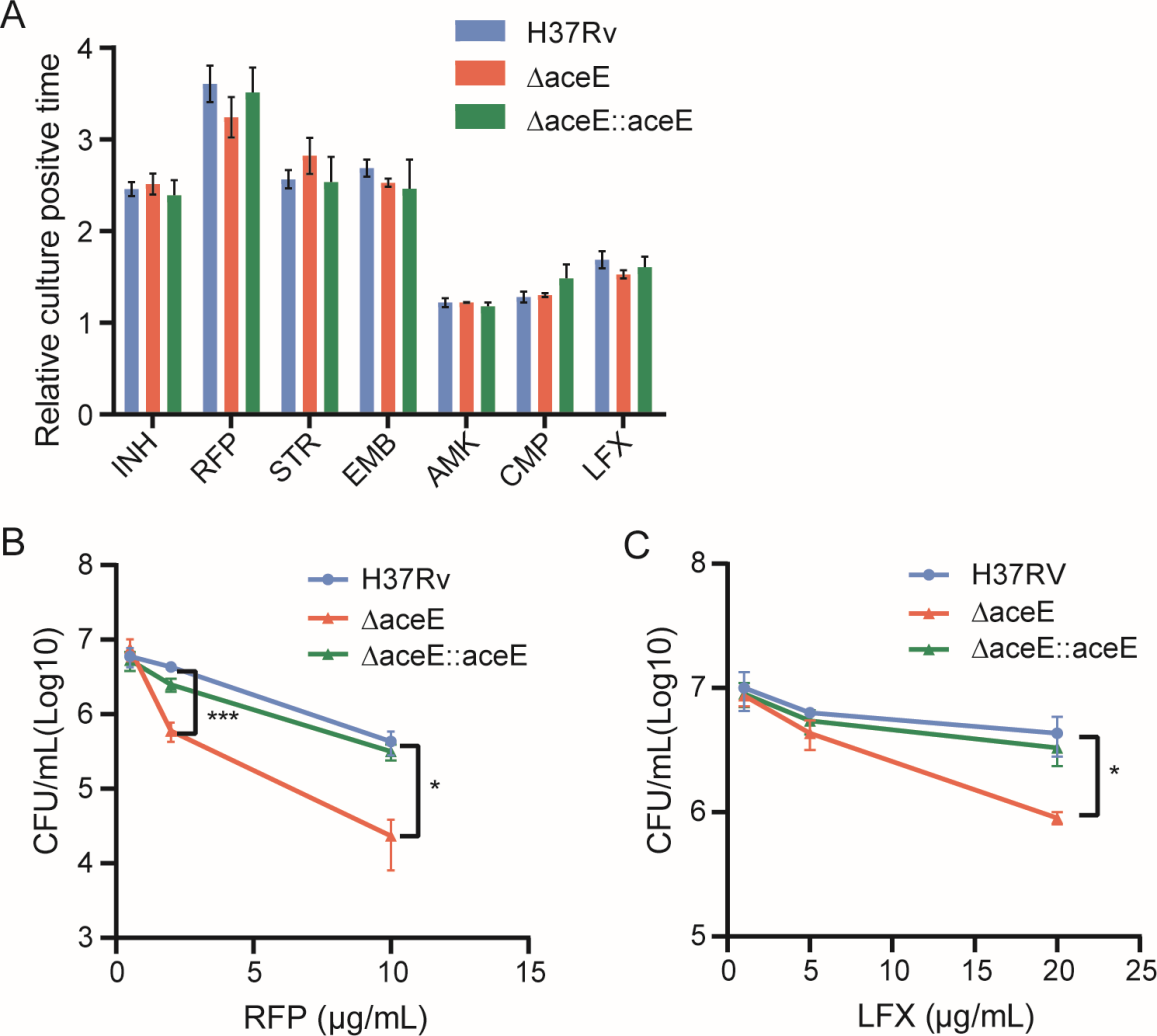
Comparison of drug susceptibility and tolerance between WT and mutant strains. (**A**) Monitor the time to positivity by the MGIT960 liquid culture system and calculate the relative positive growth time for the corresponding bacterial strain in the drug-containing medium by referring to the positive growth time of the bacterial strain in the medium without the drug. (**B**) Under the condition of rifampin (RFP) treatment with concentrations higher than MIC, the survival of bacteria was determined by CFU counting. (**C**) Under levofloxacin (LFX) treatment with concentrations higher than MIC, the survival of bacteria was determined by CFU counting. Data are means ± SD of triplicate cultures and are representative of two independent experiments. The difference between each group at each drug concentration was statistically analyzed using an unpaired *t*-test. *, *P* < 0.05; ***, *P* < 0.001. Mtb H37Rv WT strain: H37Rv; Mtb Δ*aceE* mutant: Δ*aceE*; Overexpressing the *aceE* gene in the Mtb Δ*aceE* mutant: Δ*aceE::aceE*.

Given the impact of the *aceE* gene on cell morphology and biofilm formation, we also investigated whether changes in the cell wall would affect the drug susceptibility of Mtb. We tested the growth of the Mtb Δ*aceE* strain in the presence of isoniazid (INH), rifampicin (RFP), streptomycin (STR), ethambutol (EMB), amikacin (AMK), capreomycin (CMP), and levofloxacin (LFX). Compared with the Mtb H37Rv WT strain, the inactivation of the *aceE* gene did not significantly alter the drug susceptibility of Mtb to these commonly used anti-TB drugs (Fig. 4A). However, the survival of Mtb Δ*aceE* mutant was significantly reduced at high concentrations of RFP and LFX compared to the Mtb H37Rv WT strain (Fig. 4B and C), but did not show significant defects in medium with other tested antibiotics (data not shown). This suggests that the inactivation of the *aceE* gene affects the ability of Mtb to withstand killing by some antibiotics.

### Limited impact of *aceE* gene inactivation on Mtb survival in macrophages and BALB/c mice infection

**Fig 5:**
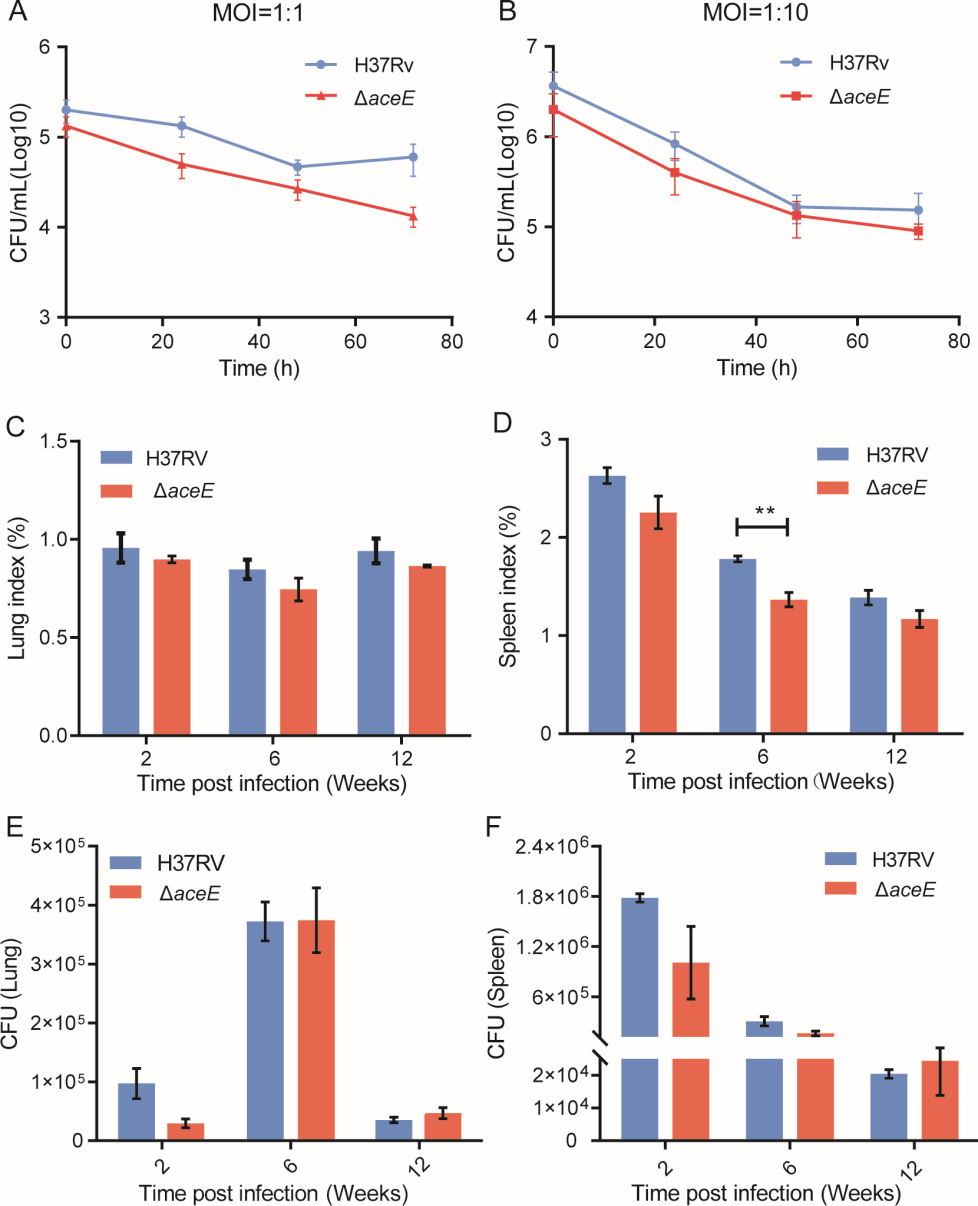
Effect of *aceE* knockout on the Mtb survival in macrophages and *in vivo* survival after infection in mice. When the MOI was 1:1 (**A**) and 1:10 (**B**), cells were harvested at different times, and the bacteria in the cells were cultured and counted. (**C**) Lung index of each group after infection. (**D**) Spleen index of each group after infection. (**E and F**) Bacterial titers from lungs and spleen of BALB/c mice infected with the indicated strains by intravenous injection. Data are means ± SD of triplicate cultures or of four mice and are representative of two independent experiments. The difference between each group was statistically analyzed using an unpaired *t*-test. **, *P* < 0.01; ***, *P* < 0.001. Mtb H37Rv WT strain: H37Rv; Mtb Δ*aceE* mutant: Δ*aceE*.

The Mtb Δ*aceE* mutant demonstrated significantly lower survival rates under acidic pH levels and sodium nitrite conditions compared to the WT strain. This observed sensitivity is noteworthy and may have important implications. Therefore, we studied the effect of this gene on the survival of Mtb cells, as well as its impact on infected mice, considering both cellular infections and mouse models. To investigate the effect of *aceE* gene inactivation on the survival of Mtb in macrophages, we carried out a macrophage infection assay using the THP-1 cell line. The results showed that the Mtb Δ*aceE* mutant was less phagocytosed by macrophages (Fig. 5A and B, 0h), suggesting Mtb Δ*aceE* mutant strains had slightly lower ability to enter the macrophages (*P* > 0.05). However, based on the slope of the bacterial clearance curve, *aceE*-deficient had little effect on the intracellular survival of Mtb (Fig. 5A and B, 24, 48, and 72 h). Next, we determined the effects of *aceE* inactivation on the *in vivo* survival of Mtb-infected mice. BALB/c mice were challenged with Mtb H37Rv WT and Mtb Δ*aceE* mutant by intravenous injection. At 2, 4, and 8 weeks of infection, the spleen of the WT infection group was significantly enlarged compared with that of the mutant strain infection group and PBS control. Still, only the 6-week comparison was significant (*P* < 0.05) (Fig. 5D). Pathological results also showed a slightly stronger inflammatory response caused by Mtb H37Rv WT infection than that of Mtb Δ*aceE* mutant (data not shown). At 2 weeks post-infection, the bacterial load in the lung and spleen in Mtb Δ*aceE* mutant was about 0.6 log_10_ CFU lower than the Mtb H37Rv WT (97,176.7 vs. 29,351; 1.78e+006 vs. 1.01e+006), but the difference was not statistically significant. Additionally, the deficiency of the *aceE* gene did not lead to a substantial reduction in CFU in the lungs and spleen in 6 and 12 weeks post-infection (Fig. 5E and F). These data demonstrated that the inactivation of the *aceE* gene reduced the inflammatory response caused by Mtb infection in mice to a certain extent, with a relatively lower invasion ability, but had a minimal effect on the survival of Mtb in BALB/c mice in the present acute infection model.

### The *aceE* gene deletion significantly alters the lipid composition of Mtb

**Fig 6:**
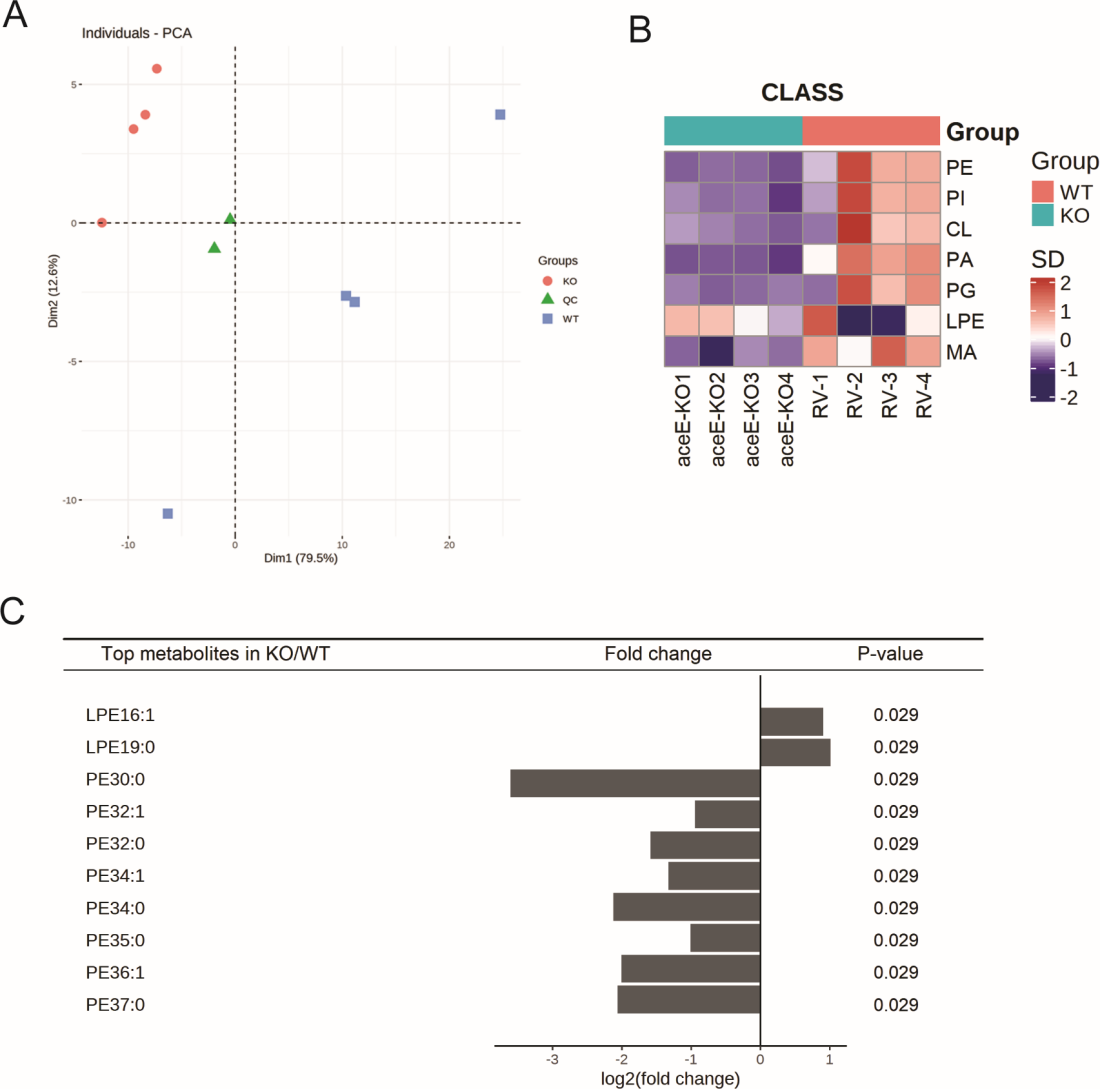
The inactivation of the *aceE* gene significantly affected the cell lipid compositions of Mtb. (**A**) The principal component analysis (PCA) of the lipid extracted from Mtb H37Rv WT and Δ*aceE* mutant strain (KO). (**B**) Lipid compositions of Mtb H37Rv WT and Δ*aceE* strain. (**C**) The top 10 significantly different lipid metabolites between Mtb H37Rv WT and the Δ*aceE* mutant strain. Mtb H37Rv WT strain: H37Rv or RV or WT; Mtb Δ*aceE* mutant: *aceE-*KO or KO.

Changes in colony morphology and biofilm formation suggested that *aceE* inactivation affected the composition of cellular lipids in the Mtb Δ*aceE* mutant. Therefore, we analyzed the lipid composition of the WT strain and Mtb Δ*aceE* mutant in the 7H9 complete medium. A total of seven types of lipids, including mycolic acids (MA), cardiolipins (CL), phosphatidylinositols (PI), phosphatidic acids (PA), phosphatidylglycerols (PG), phosphatidylethanolamines (PE), and lyso-PE (LPE), were quantified by LC-MS with the standard internal method (Fig. 6A). The results of lipidomics analysis showed that the Δ*aceE* mutant had significantly lower levels of almost all lipids except some kinds of LPE. Compared with the WT strain, the Mtb Δ*aceE* mutant had significantly lower PE, PI, CL, PA, PG, and MA and significantly upregulated LPE (16:1) and LPE (19:0) (Fig. 6B). Especially, the LPE16:1 and LPE19:0 were the significantly increased metabolites, while the PE30:0, PE32:1, PE32:0, PE34:1, PE34:0, PE35:0, PE35:0, PE36:1, and PE37:0 were the significantly downregulated metabolites in the Mtb Δ*aceE* mutant (with the lowest *P* value in the Mann-Whitney *U* test) (Fig. 6C).

### Transcriptional profile of Mtb affected by *aceE* gene inactivation

**Fig 7:**
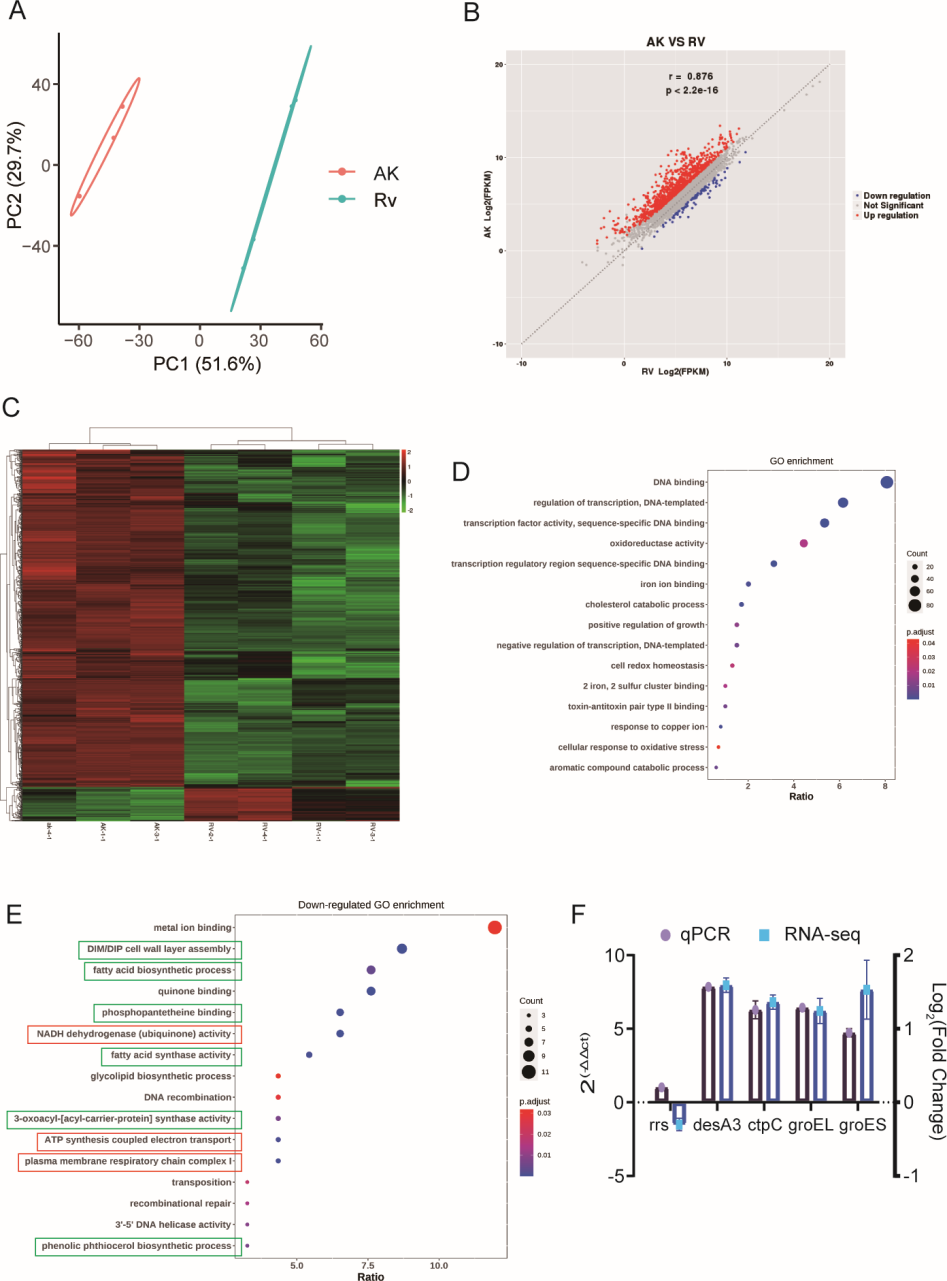
The deletion of *aceE* promotes the activation of lipid catabolism pathways. (**A**) PCA plot based on global gene transcription and expression profile for Mtb Δ*aceE* mutant (AK) and Mtb H37Rv WT strain. (**B**) DEGs between the Δ*aceE* mutant and the Mtb H37Rv WT strain. (**C**) The heatmap of the DEGs according to the adjusted *P* value. (**D**) GO analysis of DEGs in Δ*aceE* mutant and WT. (**E**) GO analysis of the downregulated genes in Δ*aceE* mutant. (**F**) Validation of the expression of randomly selected DEGs by quantification using real-time PCR. Mtb H37Rv WT strain: H37Rv or RV; Mtb Δ*aceE* mutant (AK): Δ*aceE*.

To further investigate the mechanism of the metabolic changes caused by *aceE*, we performed RNA sequencing (RNA-seq) to compare the gene transcription profiles of Mtb H37Rv and the Mtb Δ*aceE* mutant under standard growth conditions in the 7H9 complete medium. PCA was carried out to assess the quality of samples, and the data confirmed the existence of the two epi-clusters (Fig. 7A). Among the significantly differentially expressed genes, 900 were upregulated, whereas 92 were downregulated in the Δ*aceE* mutant strains (Fig. 7B and C). The differential expression of a subset of these DEGs in the ∆*aceE* mutant relative to the parental WT strain was confirmed by qPCR (Fig. 7F). To identify potential metabolism pathways that were altered by *aceE* deletion, differentially regulated genes were further classified by Gene Ontology (GO) and Kyoto Encyclopedia of Genes and Genomes (KEGG). GO enrichment analysis revealed that the differentially expressed genes were mainly enriched in functional genes such as oxidoreductase activity, cell redox homeostasis, DNA binding, cellular response to oxidative stress, regulation of growth, cholesterol catabolic process, etc. (Fig. 7D and E). Furthermore, GO analysis of the down-expressed genes showed that they were enriched in the metal ion binding and glycolipid and fatty acid synthesis processes. KEGG analysis also showed that significantly upregulated genes were enriched and associated primarily with certain lipid degradation pathways, such as steroid degradation, aminobenzoate degradation, limonene and pinene degradation, and bisphenol degradation (data not shown).

### The influence of the *aceE* gene on the growth of Mtb in a single carbon source medium

**Fig 8:**
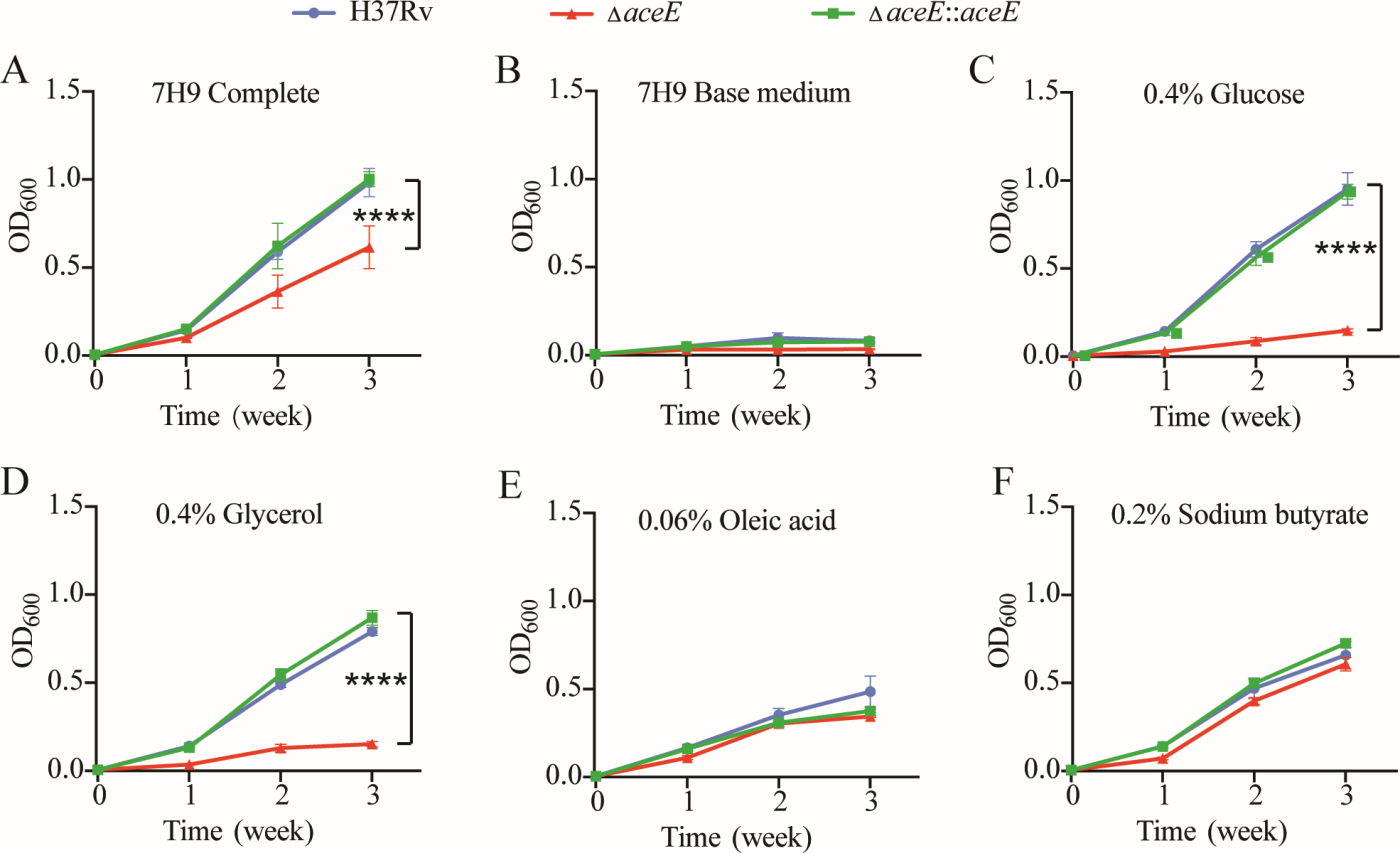
Deficiency of *aceE* impacts the optimal growth of Mtb in media that contain carbohydrate carbon sources. Bacteria were cultured in a 7H9 base medium with Tween80 (**B**) or supplemented with different carbon sources: 10% OADC (**A**), 0.4% glucose (**C**), 0.4% glycerol (**D**), 0.06% oleic acid (**E**), and 0.2% sodium butyrate (**F**). The difference between each group’s growth in the 3rd week was statistically analyzed using one-way ANOVA corrected by Tukey. ****, *P* < 0.0001. Mtb H37Rv WT strain: H37Rv; Mtb Δ*aceE* mutant: Δ*aceE*; Overexpressing *aceE* gene in Mtb Δ*aceE* mutant: Δ*aceE::aceE*.

To investigate the underlying reasons for the growth differences *ex vivo* and *in vivo*, we examined the growth of both WT and mutant strains when cultured in Sauton’s medium containing a single defined carbon source (Fig. 8). The results showed that inactivating the *aceE* gene significantly restricted the growth of Mtb when cultured in media with glucose or glycerol as the sole carbon source (Fig. 8C and D). However, this inactivation did not notably affect Mtb growth in media containing oleic acid or acetate as the sole carbon source (Fig. 8E and F). This suggests that the *aceE* gene deficiency has little impact on the metabolism of Mtb in fatty acids to some extent, with the most significant effect observed in glucose and glycerol metabolism. And we presumed that this is why the *aceE*-deficient strain did not show a clear phenotypic defect under *ex vivo* (macrophages) or *in vivo* (murine models) conditions, presumably because the prevalent carbon sources available within hosts are fatty acids rather than carbohydrates.

### The *aceE* gene knockout affects the biofilm formation of Mtb in different defined medium

**Fig 9:**
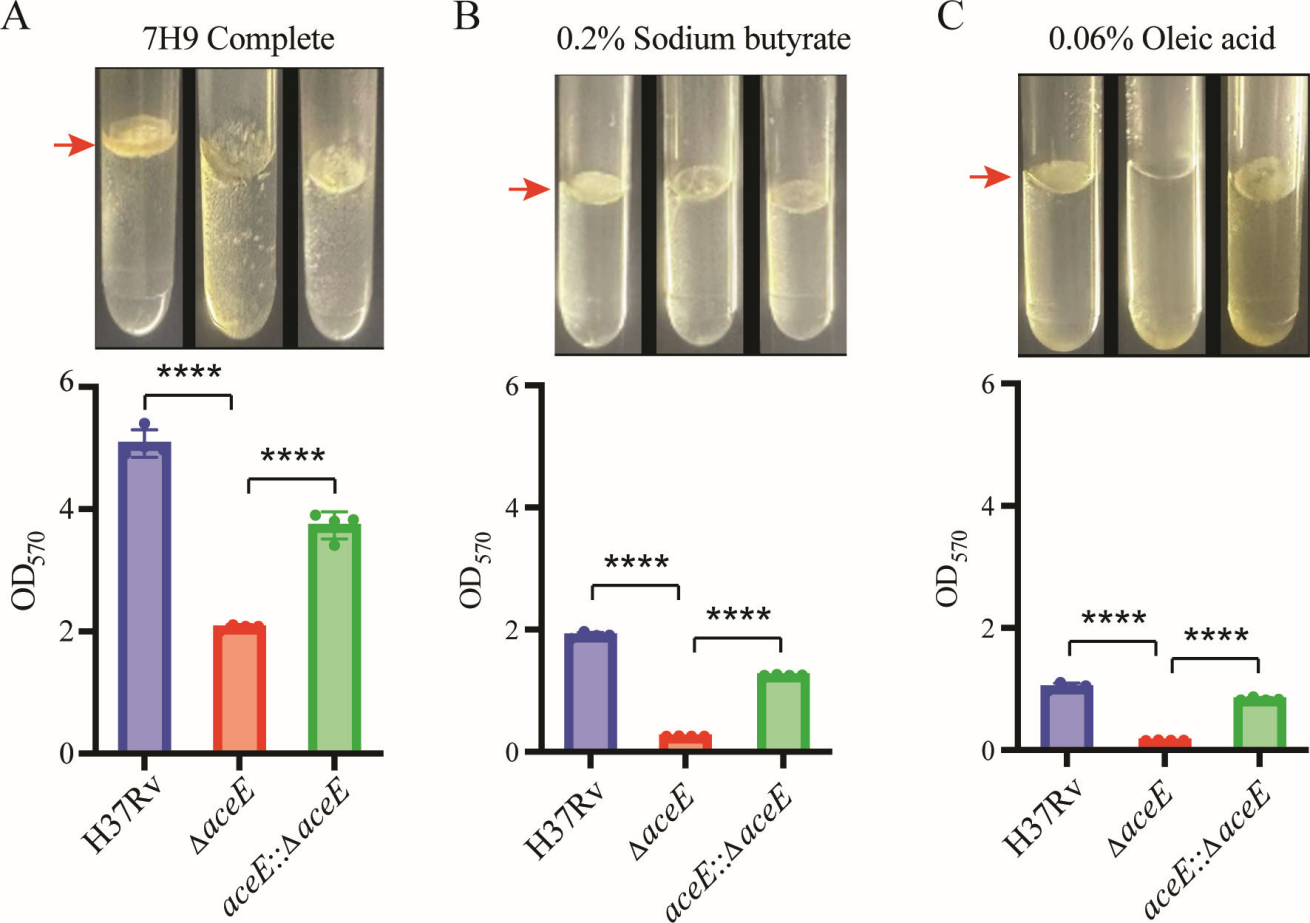
Comparison of biofilm formation between Mtb H37Rv wild-type strain and Mtb Δ*aceE* mutant strain in various media. The bacteria were cultured in three different media, all without Tween-80: the complete 7H9 medium (**A**), the 7H9 base medium with 0.2% sodium butyrate (**B**), and the 7H9 base medium with 0.06% oleic acid (**C**). The upper image shows the biofilm structure that formed at the gas-liquid interface (red arrowhead marked) of the culture tube, while the lower bar chart presents the quantitative results from crystal violet staining. The difference between each group at each drug concentration was statistically analyzed using an unpaired *t*-test. ****, *P* < 0.0001. Mtb H37Rv WT strain: H37Rv; Mtb Δ*aceE* mutant: Δ*aceE;* overexpressing *aceE* gene in Mtb Δ*aceE* mutant: Δ*aceE::aceE*.

To rule out the possibility that the biofilm formation defect of the *aceE* mutant was due to the influence of carbon source metabolic limitations, we also assessed biofilm formation in the WT strain, the *aceE* knockout strain, and the complemented strains using various media without Tween-80, including complete 7H9 medium (Fig. 9A), 7H9 base media supplemented with 0.2% sodium butyrate (Fig. 9B), and 7H9 base media supplemented with 0.06% oleic acid (Fig. 9C). Our biofilm formation analysis revealed that the Mtb Δ*aceE* mutant could not form a biofilm structure at the gas-liquid interface in all tested media. In contrast, the MtbH37Rv WT strain formed robust biofilms. Reintroducing the *aceE* gene into the Mtb Δ*aceE* mutant strain can potentially restore the phenotype observed in the WT strain. Biofilm formation plays a significant role in Mtb’s pathogenicity and drug resistance (26, 27). These findings indicate that the deletion of the *aceE* gene influences biofilm formation, which may have implications for Mtb’s pathogenicity and tolerance to treatment.

## DISCUSSION

Dehydrogenation of pyruvate is a crucial aspect of central carbon metabolism and the respiratory chain; however, its physiological role in Mtb remains poorly understood. The enzyme pyruvate decarboxylase, encoded by the *aceE* gene, is the key rate-limiting factor in converting pyruvate to acetyl-CoA. Current knowledge about *aceE* in Mtb primarily focuses on its activity in carbon metabolism. Previous studies have shown that transcription of *aceE* in the Mtb H37Rv was significantly higher in macrophages than its virulence-attenuated counterpart, H37Ra (28, 29). This suggests that *aceE* may play a role in the virulent strain’s adaptation to unfavorable conditions, which supports its persistence *in vivo*. Moreover, another study indicated that AceE can form a four-component peroxidase system with DlaT, AhpD, and AhpC, utilizing pyruvate as an electron donor in the reductase reaction. This finding reveals that Mtb may utilize some intermediate metabolites to activate a defense mechanism against reactive nitrogen species, thereby avoiding immune surveillance by the host (30). These studies collectively suggest that *aceE* is important for Mtb’s physiological metabolism and pathogenicity. In this context, we further constructed an *aceE* knockout mutant of Mtb H37Rv to investigate its role in stress tolerance and the pathogenicity of Mtb.

Consistent with other studies using *Himar* 1 transposon mutagenesis (31–33), we found that the *aceE* gene is not an essential gene for the *in vitro* growth of H37Rv; however, its inactivation did slow down the growth of Mtb *in vitro,* especially in the medium where glucose is used as the carbon source. In contrast, our previous study found no significant difference in growth between the *M. smegmatis aceE*-Tn mutant and its parental WT strain in a liquid medium (25). This discrepancy suggests that the functions of *aceE* in Mtb and its homolog in *M. smegmatis* may differ significantly. Additionally, in line with the findings of Viswanathan G et al. (34), we previously observed that the inactivation of the *aceE* gene affected the cell morphotype and biofilm formation in *M. smegmatis*. These changes were linked to reduced synthesis of alpha-MA and epoxy-MA components of mycolic acid in the cell wall (25). Our current study also showed that inactivating the *aceE* gene leads to a thinning of the Mtb cell wall and defects in biofilm formation. It has been reported that cell wall thickness increases when comparing pan-susceptible strains to multiple drug-resistant strains and extensively drug-resistant Mtb strains, suggesting an association between cell wall thickness and drug resistance (35). Biofilm formation in mycobacteria contributes to phenotypic tolerance toward harmful reagents, which allows Mtb to survive longer in the presence of anti-TB drugs (27, 36, 37). Our research revealed that the *aceE* mutant strain exhibited increased sensitivity to some specific stressors, such as acidic conditions and sodium nitrite. However, the deficiency of the *aceE* gene has no effect on the resistance of Mtb to H_2_O_2_ and SDS. It appears that either Mtb compensates for the absence of the *aceE* gene to withstand oxidative stress or the *aceE* gene has a secondary function in this response mechanism. Also, it inferred that Mtb responds to environmental pressures from peroxides and nitric oxide through different mechanisms. However, the relationship between the inactivation of the *aceE* gene and the sensitivity of Mtb to acid and nitric oxide, as well as its role in models of chronic infection, still needs further investigation. Future experiments should be designed to conduct more thorough and systematic research on this topic.

It is well established that Mtb reduces its metabolic rate to tolerate antibiotics and other drugs under low oxygen levels (12, 38). In a study by Baek et al., transposon screening methods indicated that the frequency of mutations in carbon metabolism-related genes, including *aceE*, significantly increased in the hypoxic environment (39). This suggests that the inactivation of such genes could impair Mtb’s ability to persist under hypoxia. Although *aceE*-inactivated mutants did not show altered susceptibility to the tested anti-TB drugs, they were cleared more readily by higher concentrations of RFP and LFX. This finding implies that *aceE* plays a role in maintaining the withstand killing of Mtb under these conditions.

The animal model also observed reduced invasion ability through intravenous infection of BALB/c mice (mimicking acute infection). During the acute infection, particularly at 2 weeks post-infection, the bacillary load in the lungs infected with the MtbΔ*aceE* decreased to approximately 0.6 log10 compared to the WT Mtb. Additionally, a significant reduction in spleen swelling was noted at 6 weeks post-infection, MtbΔ*aceE* compared to WT (*P* < 0.05). Although not statistically significant, clear trends indicated lower CFU counts in both the lungs and spleen at 2 weeks post-infection with MtbΔ*aceE* compared to the WT. We attribute the reduced phagocytosis by macrophage and attenuated pathology to altered cell-wall lipid composition and diminished TLR2 agonist or other PRR ligands (like LAM, which interacts with the Mannose receptor and Dectin 2, or TDMs that interact with Mincle), consistent with the hypo-inflammatory phenotype observed *in vivo*. In addition, from the defined carbon source growth assay, we observed that the *aceE* deficiency significantly impacts the growth of Mtb in glycerol or glucose medium, but has a limited effect in fatty acid medium. This observation may explain why the *aceE*-deficient strain does not exhibit a significant phenotypic defect under *ex vivo* (macrophages) or *in vivo* (murine model) conditions. This lack of impact is likely because fatty acids, rather than carbohydrates, are the predominant carbon sources available within hosts. However, the findings in the murine model warrant further investigation using a chronic infection model, as it more closely resembles natural infection conditions compared to the acute infection model.

Lipid metabolism is now recognized as a central determinant of Mtb pathogenesis and persistence (40, 41). Although fatty acids and cholesterol are considered the dominant carbon sources during infection, carbohydrates are also utilized throughout chronic disease (24, 30, 42, 43). Disruption of *aceE*, which encodes the E1 subunit of the pyruvate dehydrogenase complex, simultaneously impaired carbohydrate oxidation and rewired lipid homeostasis. Quantitative lipidomics revealed global depletion of phospholipids (PE, PI, CL, PA, and PG) and mycolic acids in the Δ*aceE* mutant, indicating reduced anabolic flux toward membrane and cell-wall lipids. Transcriptomics corroborated this observation: genes governing fatty-acid elongation and mycolate biosynthesis were downregulated, whereas transcripts encoding β-oxidation enzymes and branched pyruvate catabolism were induced. We interpret this transcriptional reprogramming as a compensatory strategy that (i) channels acetyl-CoA derived from β-oxidation into the truncated TCA cycle and (ii) diverts surplus pyruvate into alanine and glycine biosynthesis, thereby sustaining NADH production and redox balance. Such metabolic plasticity may also underlie the reduced tolerance of the *aceE* mutant to some antitubercular drugs, because prior studies have linked drug resistance to elevated fatty-acid catabolism and altered redox homeostasis (12, 44). Future studies will couple lipidomics and RNA-seq of *aceE* mutants cultured under defined carbohydrate versus fatty-acid conditions to determine how carbon-source-induced shifts in cell-wall lipid composition influence the intracellular accumulation and bactericidal activity of antitubercular drugs *in vivo*.

In conclusion, we establish that the E1 subunit of pyruvate dehydrogenase (*aceE*) is indispensable for optimal growth of Mtb, especially on carbohydrate-based media, and is a gatekeeper of cell-wall lipid homeostasis. By controlling the decarboxylation of pyruvate, AceE funnels carbon into lipid anabolism that simultaneously reinforces the barrier function of the envelope and elevates antibiotic tolerance *in vitro*. These data underscore the metabolic plasticity that enables Mtb to balance anabolic and catabolic demands in diverse host niches. Targeting this regulatory node—thereby rerouting carbon away from protective lipid biosynthesis—offers a rational strategy to lower the minimal effective dose of existing antibiotics and to shorten tuberculosis therapy.

## Data Availability

The RNA-seq data are deposited in the Genome Sequence Archive in the National Genomics Data Center, China National Center for Bioinformation/Beijing Institute of Genomics, Chinese Academy of Sciences (GSA: CRA039289) and the lipidomics mass spectrometry data are deposited in the Metabolomics Workbench (http://dx.doi.org/10.21228/M8CV9B) with the accession number ST004655.

## SUPPLEMENTAL MATERIAL

Table S1 msystems.01732-25-s0001.docx Primers used in the present study.

ASM does not own the copyrights to Supplemental Material that may be linked to, or accessed through, an article. The authors have granted ASM a non-exclusive, world-wide license to publish the Supplemental Material files. Please contact the corresponding author directly for reuse.
