# Supplementary material for: AceE affects the optimum growth and biofilm formation of Mycobacterium tuberculosis via cell wall lipid remodeling
Source: mSystems. 2026 Apr 20;11(5):e01732-25. doi: 10.1128/msystems.01732-25 (PMC13185546; doi:10.1128/msystems.01732-25)
Supplement: Table S1 — Primers used in the present study. [file msystems.01732-25-s0001.docx]

Supplemental Table S1 Primers used in the present study.

| Primers | Sequence (5’ to 3’) | Product length (bp) | Application |
| --- | --- | --- | --- |
| VLL | CCATAAATTGGAACTCAAACAGCGCAAGCGAAC | 476 | homologous recombination |
| VLR | CCATTTCTTGGAAGCGTGGCCCTGGATGAAC |  |  |
| VRL | CCATAGATTGGGCCATCGAGACCGAGAAGC | 500 | homologous recombination |
| VRR | CCATCTTTTGGGCACAGTGTCCAGCAGTTCG |  |  |
| KLL | GCGTGGATCAGATGGAATG | 325 | Identification (PCR1) |
| KLR1 | CCCTAGAGTCCTGTCCGAAATA |  |  |
| KRL | CTCGCCTTCACCTTCCTGC | 733 | Identification (PCR2) |
| KRR | TGGAGCCAACTGATTGTCGTT |  |  |
| K1s | ATGGGATGCCCTGCTGC | 685 | Identification (PCR3) |
| K1as | GTCGGGAATGATCGGGACTA |  |  |
| K1  K2 | TTACCAGACCTATAAGGC  CAGTCCGCTTTCCACGATG | 1200 | Identification (PCR4) |
